# Factors that influence resignations of radiographers employed by tertiary hospitals in the KwaZulu-Natal province, South Africa

**DOI:** 10.4314/ahs.v23i1.68

**Published:** 2023-03

**Authors:** Melisa Pillay, Pauline Busisiwe Nkosi, Maureen Nokuthula Sibiya

**Affiliations:** 1 Durban University of Technology, Faculty of Health Sciences; 2 Mangosuthu University of Technology, Division of Research, Innovation and Engagement

**Keywords:** Radiographers, staff resignation, descriptive quantitative research

## Abstract

**Background:**

The shortage of staff in Kwa-Zulu Natal (KZN) public hospitals is evident and poses a challenge to retain radiographers. Therefore, there is need to identify the factors that influence resignations of radiographers.

**Objective:**

The aim of this study was to explore the factors that influence resignations of radiographers employed by tertiary hospitals in KZN province.

**Methods:**

The study was guided by a descriptive quantitative research method. The data was collected in the form of questionnaires. A letter of information describing the details of the study was provided to the participants. All consenting participants were requested to provide a written consent prior to completing the questionnaire. The questionnaire was completed, collected and analysed. The statistical analysis test was conducted using SPSS statistic V25.

**Result:**

A response rate of 66.35% was received with 78.3% (n=108) female and 21.7% (n=30) male. The result suggested that participants unanimously agree that resignation of radiographers is precipitated by factors such as poor working conditions, unhygienic working environment and uncompetitive salaries. The result also suggested that moving from their current career in radiography to another career is precipitated by factors such as high stress level and long working hours.

**Conclusion:**

The factors identified were further analysed and results showed that the participant's personal reasons for moving from their jobs are co-related with environmental reasons. The most influential factor was stress and remuneration.

## Introduction

The existence of a global shortage of health professionals may be attributed to emigration^1^ and changing healthcare requirements^2^. Radiographers are no exception. Moreover, radiography is a challenging and rapidly advancing profession, resulting in a high global demand for radiographers^3^. The shortage of radiographers in rural and remote areas is a concern in both developed and developing countries4. In South Africa, many radiographers leave the public health sector to find employment in the private health sector or emigrate to work in other countries^4^.

Despite the suggestions by the WHO and retention policies by the Department of Health (DOH), the KZN Annual Performance Plan (APP) reported that the annual KZN radiography vacancy rate for 2016-2017 was 11%, with a turnover rate of 15%^5^. The DOH and KZN Annual reports and statistics from 2015/2016 and showed that 704 radiography posts were approved and 616 posts were filled, with a vacancy rate of 12.5%. In 2016/2017, 721 radiography posts were approved and 633 posts were filled, with a vacancy rate of 12.21%^6^. However, in 2016/2017, more posts became available, yet the vacancy rate increased by 0.29% indicating an increase in resignations in KZN public hospitals.

A study by Thambura done in 11 districts in KZN to investigate the factors impacting on the retention of radiographers demonstrated the statistics collected from the KZN DOH in 2013 and indicated a decrease in radiographers over the years 2008 to 2012. The findings of the study indicated that radiographers leave their jobs due to workload and emigration within 10 years of graduating. Radiographers leave public institutions for private practice as they seek lower workloads, better facilities and better financial rewards. There is therefore, a need to retain radiographers in this province^7^.

Thambura indicated that the factors contributing to radiographers resigning are inadequate allowances, poor working conditions, inadequate facilities and equipment, weak management support, heavy workload and limited opportunities for professional development. However, the findings suggested that radiographers remain in their jobs due to work shifts being flexible; it is also not easy to be dismissed from the public sector and there is no direct intimidation from employees or managers^7^.

The turnover of these employees may have a significant impact on an organisation's competitive advantage^8^. Employee retention is the process engaged by an organisation to retain a working environment that supports current employees and utilises various retention policies to address the needs of employees^9^. Retention in radiography is imperative as the profession has an impact on service delivery in their hospitals^10^. Many studies have analysed the factors that influence the resignations of healthcare workers^11^, ^12^, and 13. These include factors such as an aging workforce, an unfavourable working environment, recruitment policies and HR challenges in the organisation^14^. Furthermore, personal, interpersonal and organisational factors are shown to be associated with staff resignation^15^.The various factors contributing to radiographers resigning connect to Herzberg's motivation-hygiene theory, which has guided this study^14^.

Herzberg's theory was created in the 1950s to understand employee motivation, job satisfaction and to develop factors for satisfaction (motivators) and dissatisfaction (hygiene). The motivator factors include work challenges, talent recognition, increased responsibility, value of employee input in decision-making, achievement and growth, while hygiene factors include job security, pay, benefits and workplace conditions^15^. The theory demonstrates how human behaviour is influenced by these two factors^16^.

Herzberg's Motivation-Hygiene Theory is known to be an employee retention strategy to address the causes of employee turnover as explored in this study. Therefore, theory is applied to address the retention of radiographers in KZN public tertiary hospitals in this study. In this study the hygiene factors correlated to a radiographer's dissatisfaction in the workplace. These factors included pay, company policies, fringe benefits, physical working conditions, status, interpersonal relations and job security. Motivational factors correlated with recognition and achievement that enhanced radiographers to be productive, creative, committed and positively satisfied. These factors allowed radiographers to meet their psychological needs, such as recognition, a sense of achievement, growth and promotional opportunities, responsibility and meaningfulness of their work.

However, retaining a skilled workforce is significant for a health sector to function efficiently and improve their service outcomes because they have increased experience, critical knowledge and essential skills in providing continuity of service and care^17^. In Australia and New Zealand, radiographers had high reports of occupational burnout due to demographic and work-related factors^18^. The factors such as working conditions, occupation specific dispensation policy (OSD), and remuneration and career development are amongst most factors that contribute to the resignation of radiographers^18^. In African countries such as Nigeria, only 1058 radiographers were serving a high population of 150 million people. It was evident that the radiography profession was unable to attract young professionals to the career due to the poor professional image of the radiographer, created from the radiographers' lifestyles, lack of self-esteem and poor dress code^19^. In Botswana, a perceived shortage of radiographers was compounded by an increase in the need for health services due to an unbalanced distribution of staff, migration and too few trained staff^20^. Radiographers were perceived to be emigrating due to personal or family factors, ineffective healthcare, human resource management, low salaries and inadequate incentives for rural and remote services^20^.

The Ugandan healthcare sector faced a shortage of radiographers in the country and the Ugandan government intervened with a change in salaries and allowances, but the status quo remained^21^. This was due to radiographers relocating and leaving their posts in rural or remote areas. Moreover, in the United Republic of Tanzania, there is a problem with the recruitment and retention of staff^21^.

In the KZN DOH Annual Reports indicate that there is a challenge in relation to radiographers resigning in KZN public hospitals^5^, ^6^. The statistics released by the Human Resources for Health South Africa (HRMSA) in 2012 demonstrated radiographers per population of 10 000 in the private sector are higher (70.4%) than those in the public sector (29.6%). This indicates the need to retain more radiographers in the public sector^5^, ^6^. Hence, the National Department of Health (NDH) introduced policies to increase service delivery and retention of expertise in public hospitals^22^.

Literature has also shown an increase in international migration of health professionals^23^. This has deteriorated the healthcare systems in low-income countries, particularly those in sub-Saharan Africa. The migration of nurses and other health professionals from countries in sub-Saharan Africa poses a major threat to the success of the health equity^23^.

In South Africa, research was conducted in KZN (public sector) and in Gauteng (public and private sector) to review the factors impacting the retention of radiographers, job satisfaction and the lived experience of radiographers^7^,^10^, and 24. However, there has not been sufficient evident research conducted to identify the factors that influence radiographers resigning in KZN public hospitals. The current study was conducted to fulfil this gap.

## Methodology

A descriptive quantitative research method was used to identify the factors that influence resignations of radiographers employed by tertiary hospitals in KZN. In this descriptive quantitative design, the data collection was administered in the form of hard copy questionnaires. The researcher adapted the questionnaire done by author Mr M. J. Thambura^7^. The questionnaire comprised six-point Likert scale to measure the participant's responses, which were found to reach the superior parameters of the scale's reliability and validity for this study^25^.

Preceding data collection, a pilot study was conducted to improve the content validity of the questionnaire. Five radiographers participated using the questionnaire that was developed. All data were collected, and the radiographers provided the researcher with feedback in order to improve the questionnaire. The changes were implemented in the questionnaire and the validity and reliability of the construct were maintained. The statistical analysis tests were conducted using SPSS statistics V25. The completed questionnaires were collected in a sealed envelope and kept confidential by the researcher.

The participants were provided with a letter of information and consent within the ethical guidelines. Participation was voluntary with no financial incentives offered to the participants and their anonymity maintained. Radiography students, and radiography community services workers were excluded from the study. Permission to conduct the study was obtained from the KZN DOH, hospital managers from the five tertiary hospitals and radiography management. Full ethics clearance was granted by the Durban University of Technology Research Ethics Committee (IREC 080/19).

## Results

A total of 208 questionnaires were distributed according to the number of radiographers who fulfill the research criteria. Of those, a total of 138 questionnaires were completed and collected by the researcher, giving a response rate of 66.35%. Of these respondents, 21.7% (n=30) were male and 78.3% (n=108) female. They presented a range of ranks, that is, 55.8% (n=77) grade 1 radiographers, 22.5% (n=31) grade 2 radiographers, 13% (n=18) grade 3 radiographers, and 8% (11) assistant directors. With regards to years qualified, 5.1% (n=7) were <1 year qualified; 59.4% (n=82) were 1-10 years qualified; 21.7% (n=30) were >10-20 years qualified; and 13.8% (n=19) were >20 years qualified. Most respondents 54.3% (n=75) were diagnostic radiographers, 10.1% (n=14) mammography radiographers, 7.2% (n=10) nuclear medicine radiographers, 5.1% (n=7) sonographers and 23.2% (n=32) radiotherapists.

The data was analysed using nonparametric tests. A univariate analysis was applied on each item to test for significant agreement or disagreement with the statements by using the Wilcoxon signed ranks test with its central Likert score of 3.5. It is also used in the comparison of the distributions of two variables^26^. Section B of the questionnaire measured the factors that influences staff resignation and further subdivided into 2 sections.

### Factors that cause participants to move from their current job to another job in radiography either in SA or overseas

The results suggested that participants significantly agreed that moving to another radiography position in South Africa or overseas is precipitated by the following factors which all have a p<.05. The factors were uncompetitive salaries, inability to negotiate salaries, better opportunities elsewhere, poor promotion prospects and no overtime compensation. There is significant disagreement that job insecurity causes radiographers to move to another position. Therefore, job insecurity is not the reason why people leave.

**Figure 1:**
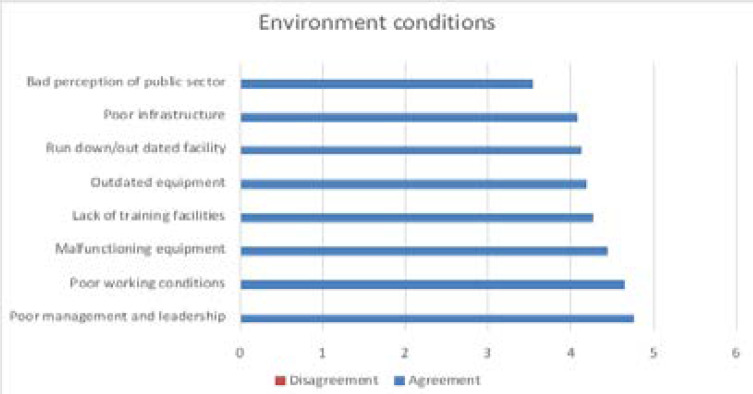
Environment factors influencing staff resignation

**Figure 2:**
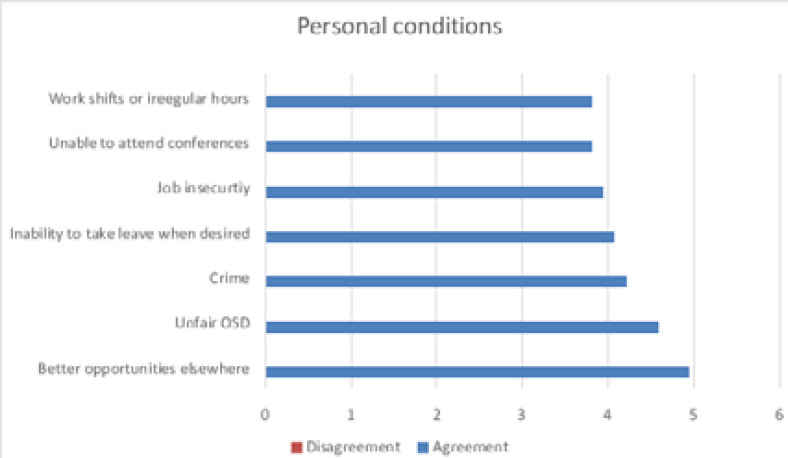
Personal factors influencing staff resignation

**Figure 3:**
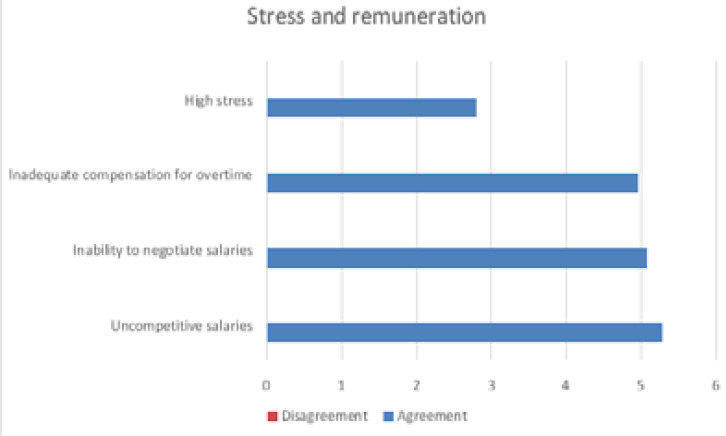
Stress and remuneration factors influencing the items of staff resignation

A factor analysis was used to check the validity and reliability of the factors. The three factors were extracted which account for 55.79% of the variance in the data environment conditions, personal conditions and stress and remuneration. The factors influencing the items of staff resignation are presented in Figure 1–3.

### Factors that cause participants to move out of radiography

The results suggested that participants significantly agreed that moving from their current career in radiography to another career is precipitated by the following factors as all the items have a p<.05. These are the factors: high stress levels, long working hours, irregular working hours, radiation hazards, poor financial rewards, limited opportunities for professional development, radiography is not highly recommended, unable to balance social and work life and job is not flexible. There is significant disagreement that radiographers do not enjoy their job, which would cause radiographers to move out of radiography.

## Discussion

The results obtained in this study focused on radiographers and managers from the five public tertiary hospitals in KZN. The three factors influencing staff resignations and retention have been highlighted in this article which is environmental conditions, personal conditions, stress and remuneration. The environment factor relates to the poor working conditions and unhygienic working environment identified in the study. Likewise, according to the SA Health^27^ hygiene is important in the prevention of the transmission of infectious diseases within healthcare facilities. Good hygiene includes the cleaning of surfaces using appropriate products; de-contamination of medical equipment and devices used in patient-care procedures. The other environment factors are related to poor infrastructure and run down/out dated facilities. Similarly, a study by Moyimane et al^28^, state that nurses in rural district hospitals in South Africa have challenges with accessing functioning medical equipment. Relative to the Moyimane et al study and this study, the respondents reported similar challenges. The critical shortage of medical equipment is due to constrained budget, low quality and poor maintenance of the few that are available. The shortage impacts negatively on nursing care, the nursing profession and the hospital. Essential infrastructure such as hospitals, accessible roads and services are important in providing quality care. There is a relationship between strong infrastructure and providing good service delivery^29^. South African public hospitals healthcare faces shortcomings with old and poorly maintained infrastructure^30^.

Similarly, a study by Nassar et al^31^, management styles play an important role in promoting workplace empowerment, commitment and job satisfaction amongst nurses in hospitals. It is also evident that the association between staff and satisfaction of working hours is significant for job satisfaction, potential burnout, contentment with work schedule flexibility and staff retention^32^.

The stress and remuneration factors relate to high stress levels, uncompetitive salaries, inability to negotiate salaries and inadequate compensation for working overtime. A similar scenario of salary stagnation with no significant increases in salaries arose with the OSD policy for nurses^33^. However, the researcher acknowledges that there could be many more factors contributing to staff retention within the public health sector but is beyond the scope of this article. These results correlate to the findings in the study done on factors affecting job satisfaction amongst radiographers in Gauteng^7^ and the lived experiences of radiographers in Gauteng, South Africa^10^. These are amongst the factors that the KZN Department of Health is required to address in an endeavour to limit resignations, influence the retention and desirability of radiographers within the KZN public sector.

## Conclusion

It was evident that most of the respondents in the study, were not satisfied with the remuneration, career advancement and the government OSD policy. These factors had a significant correlation with intent to leave. Furthermore, participants raised concerns regarding infrastructure and work environment. The factors that were identified were correlated to the environment, personal stress and remuneration conditions. The evidence from the vacancy rate statistics presented a need to explore the retention problems in KZN public hospitals.

## Recommendations

Retention of radiographers

• The KZN DOH should consider reviewing and increase the budget to repair malfunctioning equipment or purchase new equipment.

• Radiography posts are occasionally frozen, which has led to severe staff shortages. Therefore, managers The DOH should fill vacant positions in each financial year to avoid increased workload due to staff shortage.

Future research

• Radiography leadership and managerial qualities should be explored to identify a management style suitable for the managerial position and current workforce.

• Explore the coping mechanism used by radiographers to deal with work related stress and their intent to leave.

• Grade 1 radiographers, also known as the younger radiographers, showed the greatest intent to leave. Hence, this problem needs to be further investigated.
